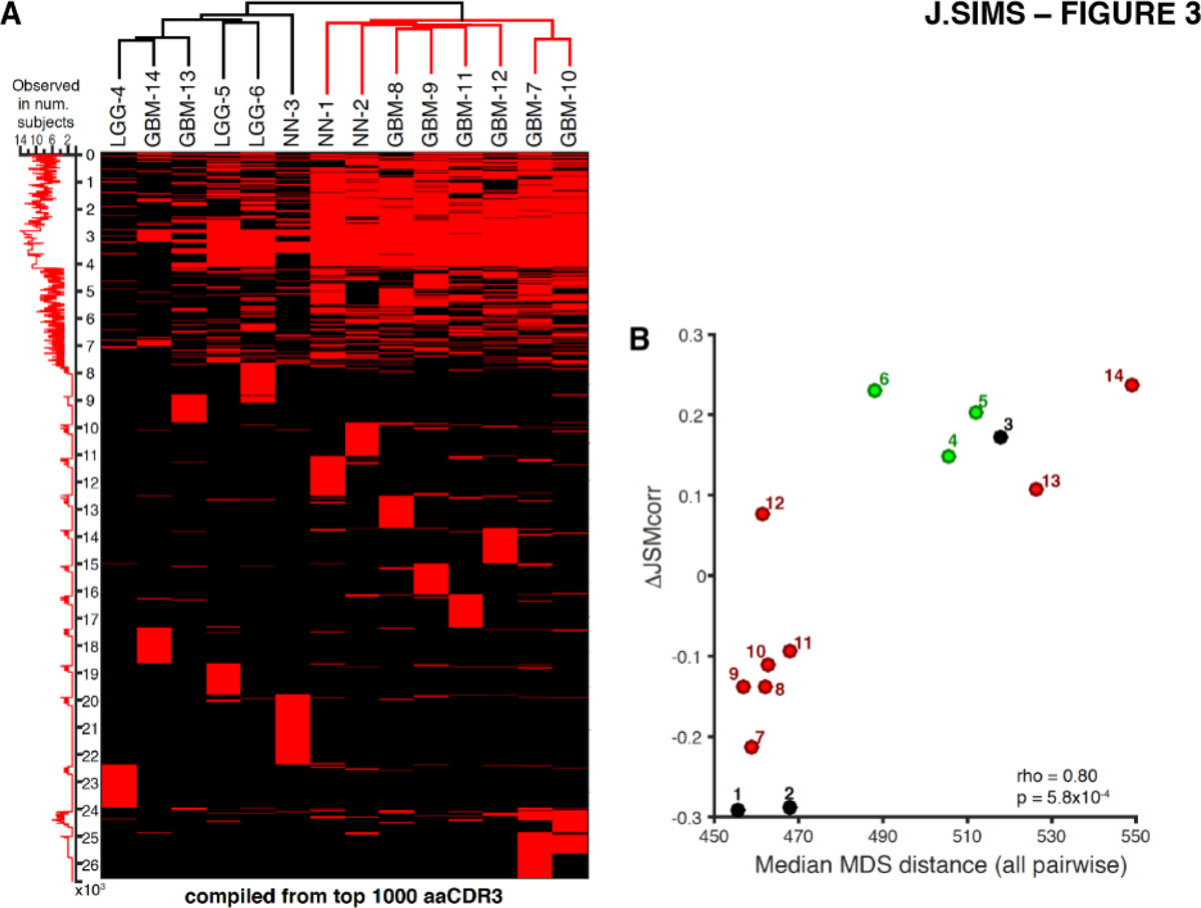# Precision immunophenotyping by high-throughput TCR sequencing in human glioma

**DOI:** 10.1186/2051-1426-3-S2-P387

**Published:** 2015-11-04

**Authors:** Jennifer Sims, Boris Grinshpun, Yaping Feng, Timothy Ung, Justin Neira, Jorge Samanamud, Peter Canoll, Yufeng Shen, Peter Sims, Jeffrey Bruce

**Affiliations:** 1 Dept. of Neurological Surgery, Columbia University, New York, NY, USA; 2 Dept. of Systems Biology, Columbia University, New York, NY, USA; 3 Waksman Institute, Rutgers University, New Brunswick, NJ, USA; 4 School of Medicine, University of Colorado, Denver, Aurora, CO, USA; 5 Dept. of Pathology & Cell Biology, Columbia University, New York, NY, USA

## 

Immunotherapy for glioblastoma (GBM) is the subject of numerous clinical trials, given the potential for the adaptive immune response to combat this diffusely infiltrating tumor. However, rational application of immunotherapy to these tumors is challenging because of the peculiar immune privilege of the brain and the molecular heterogeneity of glioma antigens. Little is known about patient-to-patient variability in the potential to generate anti-glioma immune responses of their systemic and local T cell populations.

We conducted a foundational study of the population-wide characteristics of both the peripheral blood and local, tissue-infiltrating T lymphocytes (TILs) of glioma patients using TCRseq. Through reverse transcription and pan-repertoire amplification of the TCR-alpha and -beta chains we generated TCRseq libraries from matched surgical tissue and peripheral blood cells. Using peripheral blood samples from primary GBM, low-grade glioma, and non-tumor patients (N=14), we define new immunophenotypes using statistical metrics based on information theory – specifically, the clonality associated with antigen-binding function in the TCRs of TIL, and the antigen-binding associated divergence of the TIL population from the blood. We found that diversification of this repertoire was characteristic of high-grade tumors, and correlated strongly with the activation of specific immune and inflammatory pathways. Furthermore, we found strong correlation between usage of a subset of TCR sequences in patients' peripheral blood and the divergence of their TIL population from the peripheral repertoire. We anticipate that these immunophenotypes will be key to monitoring and predicting response to anti-glioma vaccines and immunotherapy, and understanding the immunological mechanisms determining their efficacy.

**Figure 1:**
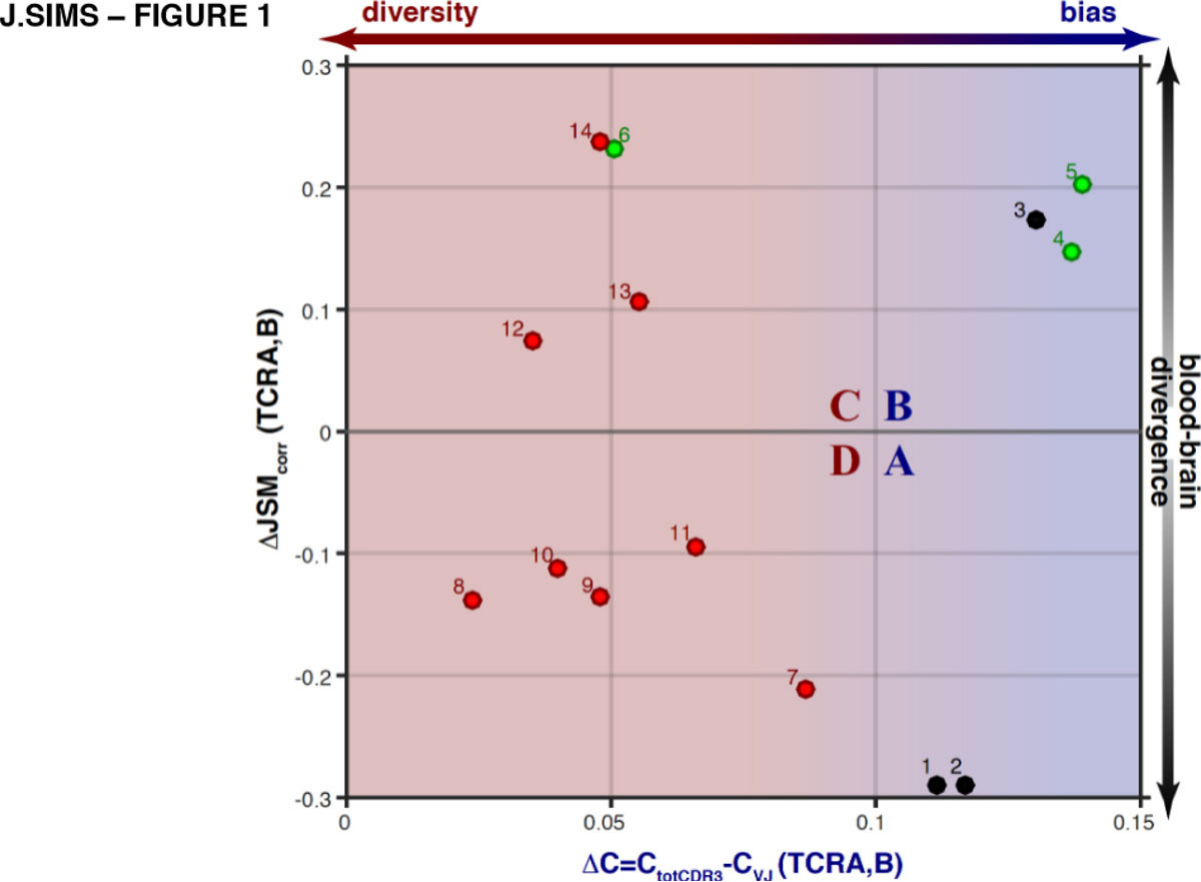


**Figure 2:**
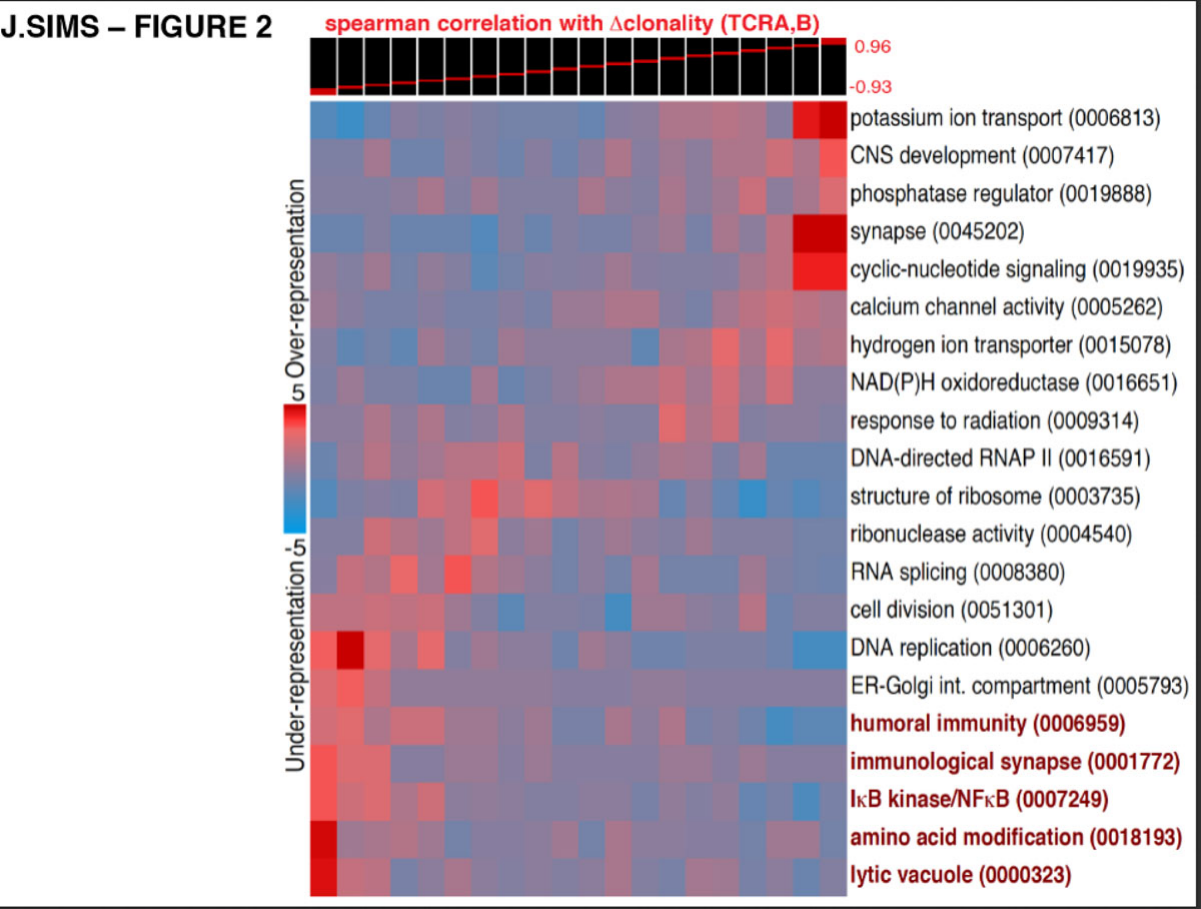


**Figure 3:**